# Mechanochemical Synthesis of TiO_2_ Nanocomposites as Photocatalysts for Benzyl Alcohol Photo-Oxidation

**DOI:** 10.3390/nano6050093

**Published:** 2016-05-18

**Authors:** Weiyi Ouyang, Ewelina Kuna, Alfonso Yepez, Alina M. Balu, Antonio A. Romero, Juan Carlos Colmenares, Rafael Luque

**Affiliations:** 1 Department of Organic Chemistry, University of Cordoba, Edificio Marie Curie(C-3), Ctra Nnal IV-A, Km 396, Cordoba E14014, Spain; qo2ououw@uco.es (W.O.); z22yegaa@uco.es (A.Y.); qo2balua@uco.es (A.M.B.); qo1rorea@uco.es (A.A.R.); 2 Institute of Physical Chemistry Polish Academy of Sciences (PAS), Kasprzaka 44/52, Warsaw 01-224, Poland; ekuna@ichf.edu.pl (E.K.); jcarloscolmenares@ichf.edu.pl (J.C.C.)

**Keywords:** TiO_2_, magnetically separable photocatalysts, selective photo-oxidation, mechanochemical synthesis, ball mill

## Abstract

TiO_2_ (anatase phase) has excellent photocatalytic performance and different methods have been reported to overcome its main limitation of high band gap energy. In this work, TiO_2_-magnetically-separable nanocomposites (MAGSNC) photocatalysts with different TiO_2_ loading were synthesized using a simple one-pot mechanochemical method. Photocatalysts were characterized by a number of techniques and their photocatalytic activity was tested in the selective oxidation of benzyl alcohol to benzaldehyde. Extension of light absorption into the visible region was achieved upon titania incorporation. Results indicated that the photocatalytic activity increased with TiO_2_ loading on the catalysts, with moderate conversion (20%) at high benzaldehyde selectivity (84%) achieved for 5% TiO_2_-MAGSNC. These findings pointed out a potential strategy for the valorization of lignocellulosic-based biomass under visible light irradiation using designer photocatalytic nanomaterials.

## 1. Introduction

Photocatalysis has been considered as one of the most environmentally friendly and promising technologies owing to advantages such as being clean, efficient, cost-effective, and energy-saving [1,2,3] Typical applications of photocatalysis are conversion of CO_2_ to fuels and chemicals [4,5,6,7,8], self-cleaning surfaces [9,10], disinfection of water [11,12], oxidation of organic compounds [13,14,15], and production of hydrogen from water splitting [16,17,18,19]. In this regard, different types of heterogeneous photocatalysts have been extensively reported, including metal oxide nanoparticles, composite nanomaterials, metal-organic frameworks, plasmonic photocatalysts, and polymeric graphitic carbon nitride [3,4].

Among these different types of photocatalysts, TiO_2_ has been extensively investigated and is one of the most widely used in the aforementioned applications due to its excellent photocatalytic activity, high thermal and chemical stability, low cost, and non-toxicity [20,21]. However, in spite of its advantages, the main drawback of TiO_2_ in photocatalysis relates to the large band gap (3.2 eV) for its anatase crystalline phase which restricts its utilization to ultraviolet (UV) irradiation (λ < 387 nm), with UV irradiation comprising less than 5% of the solar energy. Therefore, it is very important to extend the photocatalytic activity of TiO_2_ nanocatalysts under visible light to profit from abundant solar energy. Various approaches have been developed to improve the photoactivity of TiO_2_ by lowering the band-gap energy and delaying the recombination of the excited electron-hole pairs, *i.e.*, cationic [22,23] and anionic [20,24,25] doping, dye photosensitization, deposition of noble metals. Photocatalysts doped with noble metals can improve their photoactivities, but with limitations for large scale applications. Importantly, the design of photocatalysts featuring magnetic separation has not been considered to a large extent despite the obvious advantages of separation and recycling for magnetically-separable heterogeneous photocatalysts [26]. Conventional methods for heterogeneous catalyst recovery, such as filtration, centrifugation, *etc.*, are either time consuming or costly, while the enhanced magnetically-separable properties of the heterogeneous catalyst can exceed these limitations. In recent years, photocatalysts with TiO_2_ coated on magnetic particles have been reported by many researchers [27,28,29], which showed enhanced photocatalytic activities and feasible separation by applying external magnetic field. Ojeda *et al.* reported a maghemite/silica nanocomposites, which were also magnetically separable [30], followed by a report on the incorporation of TiO_2_ on maghemite/silica nanocomposites under ultrasounds which exhibited excellent photocatalytic performance in the selective oxidation of benzyl alcohol [31].

The selective oxidation of alcohols to the corresponding carbonyl compounds accounts for one of the most significant transformations in organic chemistry. Particularly, the conversion of benzyl alcohol (BA) to benzaldehyde (BHA) has attracted extensive attention, since benzaldehyde is widely applied in food, pharmaceutical, and perfumery industries and as building block in other chemical industries. Recently, the photocatalytic oxidation of benzyl alcohol to benzaldehyde has been reported using different catalysts and chlorine-free benzaldehyde with high selectivity, with respect to the traditional syntheses-either by benzyl chloride hydrolysis or via toluene oxidation [15,26,32].

In continuation with research efforts from the group related to the design of advanced nanomaterials for (photo)catalytic processes, we aimed to synthesize an advanced magnetically-separable nanophotocatalyst (TiO_2_-MAGSNC) using a simple one-pot mechanochemical method under ball mill. A widely-reported porous support (SBA-15) was utilized as support, together with an iron precursor and propionic acid to obtain a magnetic phase able to provide magnetically-separable features to the catalyst. A high-energy ball milling process was applied in this work which could provide small nanoparticle sizes as well as a highly homogeneous crystalline structure and morphology. TiO_2_-MAGSNC catalysts were found to be photoactive with a high selectivity in the selective oxidation of benzyl alcohol to benzaldehyde.

## 2. Experimental

### 2.1. Synthesis of TiO_2_/MAGSNC Photocatalysts

SBA-15 silica was prepared using the procedure reported by Bonardet *et al.* [33] Different amounts of titanium precursor were used to obtain various contents of TiO_2_ (0.5, 1.0, 2.0, 5.0 wt %) on the catalysts. Titanium incorporation was subsequently achieved by a simple mechanochemical method in a planetary ball mill under previous optimized conditions [34]. In detail, Pluronic P123 surfactant (Sigma-Aldrich Inc., St. Louis, MO, USA) (8.0 g) was dissolved in deionized water (260 mL) and HCl (Panreac Química S.L.U., Barcelona, Catalonia, Spain) (12 M, 40 mL) under vigorous stirring, at 40 °C for 2 h. Upon complete dissolution, 7 g of tetraethyl orthosilicate (TEOS) (Sigma-Aldrich Inc., St. Louis, MO, USA) were added dropwise to the above solution. The mixture was stirred at 40 °C for 24 h, followed by hydrothermal treatment at 100 °C for 48 h in an oven. The white solid was separated from the solution by filtration and dried at 60 °C. The template was removed by calcination at 600 °C for 8 h. Different amounts (13, 59, 188 and 661 μL) of titanium isopropoxide (Sigma-Aldrich Inc., St. Louis, MO, USA), 1.34 g Fe(NO_3_)_3_·9H_2_O (Merck, Darmstadt, Hesse, Germany), 0.5 g SBA-15 and 0.25 mL propionic acid (Panreac Química S.L.U., Barcelona, Catalonia, Spain) were added to a 125 mL reaction chamber with eighteen 10 mm stainless steel balls and then ground in a Retsch PM-100 planetary ball mill (350 rpm, 10 min) (Retsch GmbH, Haan, North Rhine-Westphalia, Germany). Materials calcination was performed at 400 °C (heating rate 3 °C/min) for 5 h in a furnace under an oxygen deficient atmosphere (static air). MAGSNC sample was synthesized under same conditions without adding titanium isopropoxide. All chemicals were used as received.

### 2.2. Characterization of the TiO_2_-MAGSNC Photocatalysts

The crystal phase structures of TiO_2_-MAGSNC samples were examined by powder X-ray diffraction (XRD) measurements performed in a Bruker D8 DISCOVER A25 diffractometer (Bruker Corporation, Billerica, MA, USA) equipped with a vertical goniometer under theta-theta geometry using Ni filtered Cu Kα (λ = 1.5418 Å) radiation and operated at 40 KeV and 40 mA. Wide angle scanning patterns were collected from 10° to 80° with a step size of 0.01° and counting time of 500 s per step.

Textural properties of the samples were determined by N_2_ physisorption using a Micromeritics ASAP 2020 automated system (Micromeritics Instrument Corporation, Norcross, GA, USA) with the Brunauer-Emmet-Teller (BET) and the Barret-Joyner-Halenda (BJH) methods. Prior to adsorption measurements, samples were degassed under vacuum (0.1 Pa) for 4 h at 300 °C.

A UV/VIS/NIR spectrophotometer Jasco V-570 (JASCO international Co., Ltd., Hachioji, Tokyo, Japan) equipped with an integrating sphere was used to record Ultraviolet-Visible (UV-VIS) diffuse reflectance spectra. The baseline was obtained with SpectralonTM (poly(tetrafluoroethylene) as a reference material. The Kubelka-Munk method was utilized (from diffuse reflectance spectra) to determine the band gap function. Function *f*(*R*) was calculated from the following equation:

(1) $$f\left(R\right)=\frac{{\left(1-R\right)}^{2}}{2R}$$

while E_g_ was calculated from (*f*(*R*)hν)^1/2^ *versus* hν plots.

X-ray photoelectron spectroscopy (XPS) measurements were carried out with a VG Scientific photoelectron spectrometer ESCALAB-210 (Thermo Scientific, Waltham, MA, USA) with Al Kα radiation (1486.6 eV) from an X-ray source, operating at 15 kV and 20 mA. Survey spectra in the energy range from 0 to 1350 eV with 0.4 eV step were recorded for all the samples. High resolution spectra were recorded with 0.1 eV step, 100 ms dwell time and 25 eV pass energy. A ninety degree take-off angle was employed in all measurements. Curve fitting was carried out using the CasaXPS software (Casa Software Ltd., Cheshire, England, UK), which each component of the complex envelope is described as a Gaussian–Lorentzian sum function; a constant 0.3 (±0.05) G/L ratio was used. The background was fitted using a nonlinear Shirley model. Measured transmission function and Scofield sensitivity factors have been employed for quantification purposes. An aromatic carbon C 1s peak at 284.5 eV was used as the reference of binding energy.

Scanning electron microscopy images were recorded with a JEOL JSM-6300 scanning microscope (JEOL Ltd., Akishima, Tokyo, Japan) equipped with Energy-dispersive X-ray spectroscopy (EDX) at 20 kV. An Au/Pd coating was employed to analyze samples on a high-resolution sputtering SC7640 instrument (Quorum Technologies Ltd., Lewes, England, UK) (up to 7 nm thickness) at a sputtering rate of 1.5 kV per minute.

FEI Tecnai G2 (FEI Tecnai, Hillsboro, OR, USA) fitted with a Charge-coupled Device (CCD) camera for ease and speed of use was applied to record the transmission electron microscopy (TEM) images of the synthesized TiO_2_-MAGSNC samples at the Research Support Service Center (SCAI) from Universidad de Cordoba. The resolution of the equipment is around 0.4 nm. Prior to the recording, samples were prepared by suspension in ethanol, assisted by sonication and followed by deposition on a copper grid.

The magnetic susceptibility was measured at low frequency (470 Hz) using a Bartington MS-2 (Bartington Instruments Ltd., Witney, England, UK), at room temperature.

### 2.3. Photocatalytic Experiments

**Figure 1:**
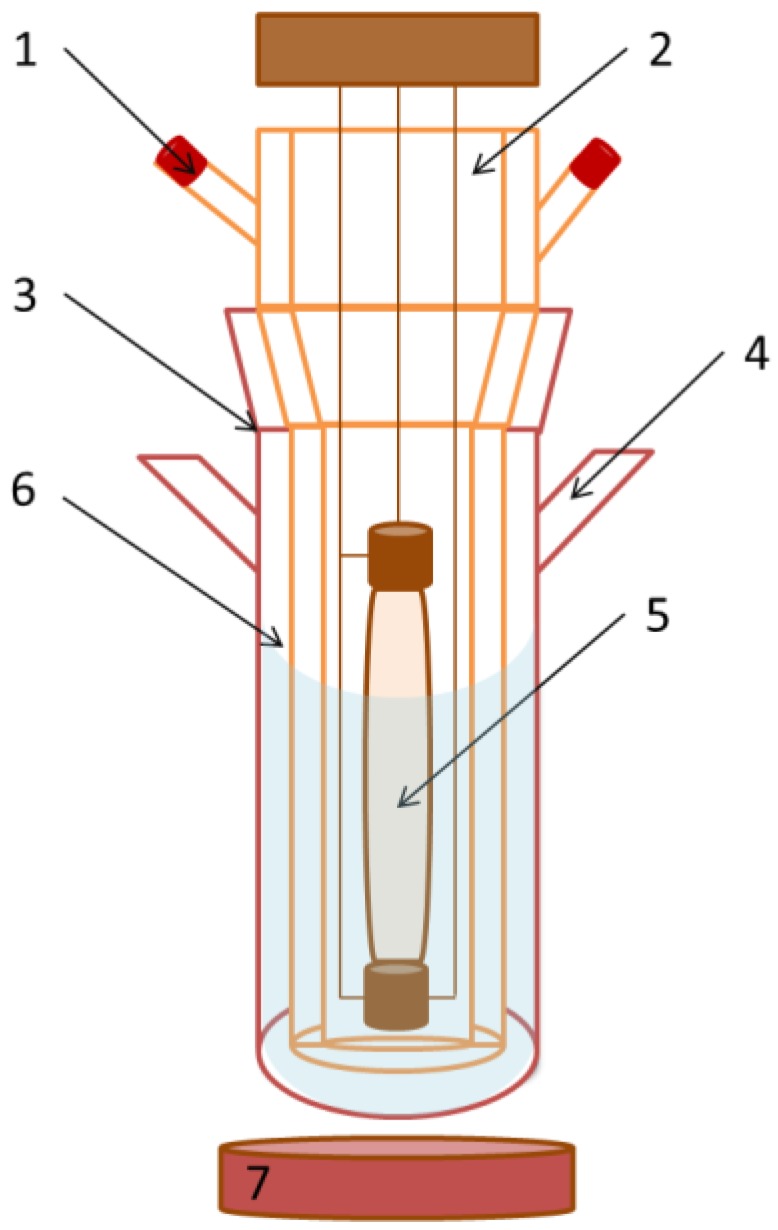
Reaction system: (1) lamp cooling system; (2) double-walled immersion well reactor; (3) photoreactor; (4) port for taking samples; (5) 125 W ultraviolet (UV) lamp; (6) mother solution; and (7) magnetic stirrer.

A Pyrex cylindrical double-wall immersion well reactor equipped with medium pressure 125 W mercury lamp (λ = 365 nm), which was supplied by Photochemical Reactors Ltd. UK (Model RQ 3010), (Reading, UK) was used in all the catalytic reactions (Figure 1). The distance between the light source and reaction media was *ca.* (*ca.*: abbreviation of circa) 10 nm and irradiance of the light source reached 1845.6 W/m^2^. Magnetic stirring with a speed of 1100 rpm was utilized in the batch reactor to obtain a homogenous suspension of the TiO_2_-MAGSNC photocatalysts. The reaction temperature was established at 30 °C. 1.5 mM benzyl alcohol (Sigma-Aldrich Inc., St. Louis, MO, USA) was prepared in acetonitrile (Sigma-Aldrich Inc., St. Louis, MO, USA) medium. Experiments were performed from 150 mL of the mother solution and 1 g/L of catalyst concentration for 4 h under UV light and air bubbling conditions (25 mL/min). In order to equilibrate the adsorption-desorption over the photocatalyst surface, the reaction solution was left in the dark for 30 min before each reaction. Samples were periodically withdrawn (*ca.* 1 mL) from the photoreactor at different times and filtered off (0.20 μm, 25 mm, nylon filters). The concentration of model compound was determined by a high performance liquid chromatography (HPLC, Waters Model 590 pump) (Waters Limited, Hertfordshire, UK) equipped with a dual absorbance detector (Waters 2487) and the SunFire™ C18 (3.5 μm, 150 mm length, 4.6 mm inner diameter) column provided by Waters. The mobile phase was Milli-Q water/acetonitrile/methanol in the volumetric ratio of 77.5:20:2.5 with 0.1% of H_3_PO_4_ (Sigma-Aldrich Inc., St. Louis, MO, USA). We used isocratic elution at a flow rate of 1 mL/min. The injection volume was 10 μL. TiO_2_ P25 (approx. 80% anatase and 20% rutile) is a commercial catalyst purchased from Evonik Industries (Evonik Industries AG, Essen, Germany) and used as comparison here.

## 3. Results and Discussion

**Figure 2:**
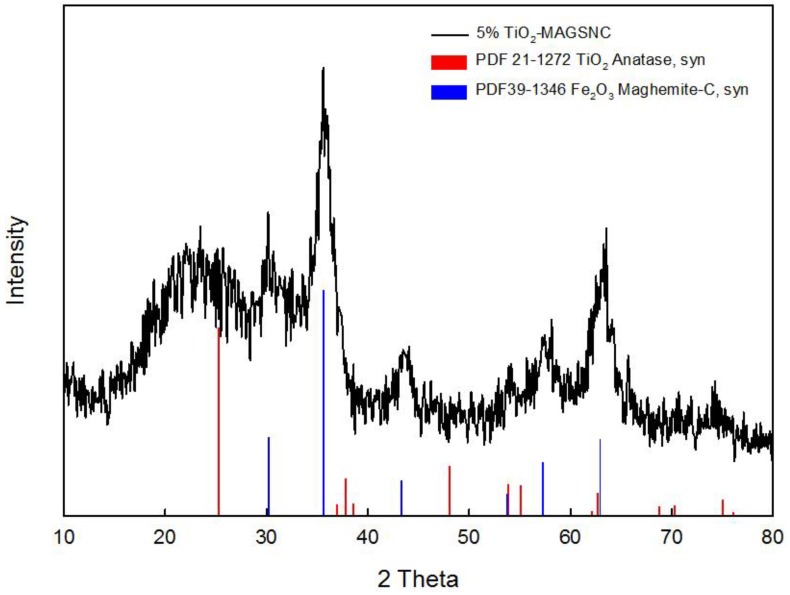
X-ray diffraction (XRD) pattern of 5% TiO_2_-magnetically-separable photocatalysts (MAGSNC) photocatalysts. (PDF 21-1272 and PDF 39-1346 are the card numbers for the crystalline structures in the data base, while Anatase, syn and Maghemite-C, syn are the corresponding structure names.)

XRD analysis was performed to investigate the crystal phase of the synthesized TiO_2_-MAGSNC nanocomposites. The XRD pattern of a representative sample (5% TiO_2_-MAGSNC) is shown in Figure 2 The mean observed peaks (2θ = 35.6°) could be assigned to the presence of a magnetic phase (in principle γ-Fe_2_O_3_, although the presence of a magnetite phase cannot be completely ruled out) while titania peaks were not obvious due to the low titanium loading on the supports; hence, particle size could not be worked out from these data. By applying the Scherrer equation, iron oxide nanoparticle sizes can be calculated to be *ca.* 14 nm. Results from XRD pattern also suggested that our simple mechanochemical protocol can successfully lead to the formation of magnetically-separable nanocomposites, as further supported with subsequent characterization techniques.

**Figure 3:**
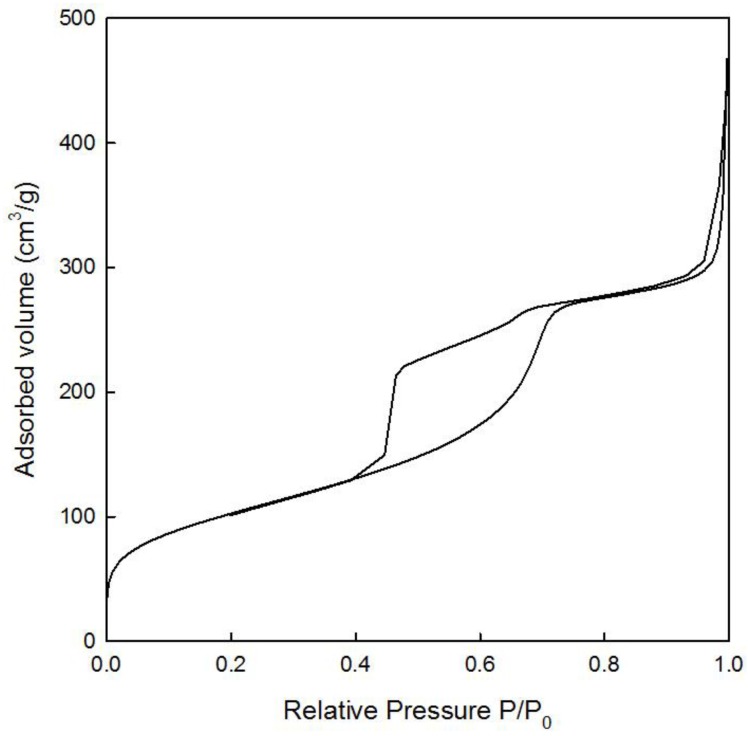
N_2_ absorption-desorption isotherm of 5% TiO_2_-MAGSNC photocatalysts. P: partial vapor pressure of adsorbate gas in equilibrium with the surface at 77.4 K; P_0_: saturated pressure of adsorbate gas.

N_2_ absorption-desorption isotherms were used to evaluate the textural properties of the TiO_2_-MAGSNC samples with different content of TiO_2_. The isotherms (Figure 3) matched the characteristic type IV isotherm profile indicating these samples are essentially mesoporous in nature. In comparison to commercial titanium oxide (59 m^2^·g^−1^) our materials possess significantly higher surface area (generally 400–500 m^2^·g^−1^), without any significant changes in terms of textural properties with respect to those of the parent MAGSNC, probably due to the low titania loading. These could also be observed in TEM images. Pore volumes in the 0.40–0.45 mLg^−1^ range and diameters typical of the parent SBA-15 material (*ca.* 6 nm) were also obtained.

**Figure 4:**
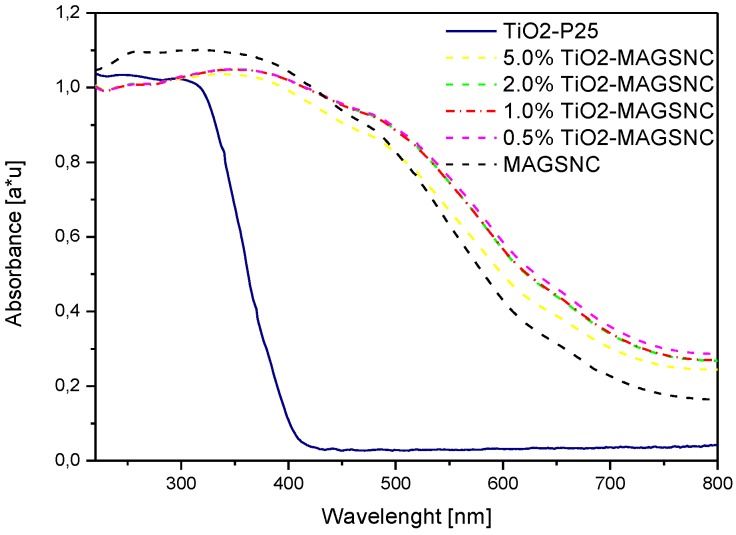
Diffuse reflectance (DR) Ultraviolet-Visible (UV-VIS) absorption spectra of different TiO_2_-MAGSNC photocatalysts. P25: pure commercial TiO_2_ from Evonik Industries.

Diffuse reflectance (DR) UV-VIS spectroscopy was used to record the optical properties of the samples. UV-VIS adsorption spectra of TiO_2_-MAGSNC samples are shown in Figure 4, which showed extensions of absorption band into the visible region for all catalysts. Significant enhancement of light absorption of all samples was achieved at a wavelength of around 700 nm, when comparing to those of pure commercial TiO_2_ (P25, 386 nm). The extension of light absorption of the synthesized catalysts into the visible range was probably resulting from the presence of the photocatalytic composite, iron oxide phase, on the MAGSNC supports. As a result of the extension of light absorption into the visible light range, better utilization of the abundant solar energy might be possible.

The band gaps of synthesized TiO_2_-MAGSNC were calculated, based on the Kubelka-Munk function (Table 1), to be in the 1.62 to 1.67 eV range. These extraordinary low values are derived from the iron oxide phase formed during ball mill in the synthetic stage as a result of the mechanochemical process [30,34], which only slightly decrease upon titanium incorporation. With Fe^3+^ radius (0.64 Å) close to that of Ti^4+^ (0.68 Å), the incorporation of Fe^3+^ into the TiO_2_ crystal lattice during synthesis may also take place [35]. The proposed one-pot synthesis procedure might facilitate the incorporation of Fe^3+^ and formation of heterojunctions between TiO_2_ and iron oxide phases during the transformation of titanium precursor to TiO_2_ which might favor the charge separation in the catalysts and further improve the photocatalytic activity.

**Figure 5:**
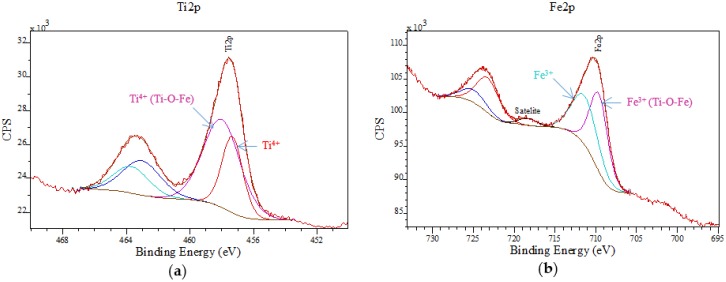
X-ray photoelectron spectroscopy (XPS) spectra of 5% TiO_2_-MAGSNC photocatalysts: (**a**) Ti 2p; and (**b**) Fe 2p.

In order to analyze the chemical states of the prepared samples, XPS spectra were also recorded. Figure 5a depicts binding energies (BEs) of *ca.* 463.3 and 457.5 eV for Ti 2p3/2 and Ti 2p1/2, respectively, characteristic of the Ti^4+^ cation with a 5.8 eV spin orbit splitting. The fitting peak with higher binding energy arises from the Ti^4+^ species in a Ti–O–Fe structure. Electrons can be induced by transfer from Ti^4+^ to Fe^3+^ in the Ti–O–Fe bond due to the electronegativity difference between Ti^4+^ (1.54) and Fe^3+^ (1.83), which makes Ti^4+^ species potentially less electron-rich (and Fe^3+^ more electron-rich), resulting in the increase of BE for Ti^4+^ species and decrease of BE for Fe^3+^ [36]. Peaks at a binding energy of 723.8 (Fe 2p1/2) and 710.2 eV (Fe 2p3/2) also correlated well to typical signals of Fe^3+^ from Fe 2p in Figure 5b, which confirmed the presence of such species in the nanocomposites, in good agreement with XRD results. Despite the stability of the hematite phase (as most thermodynamically stable at temperature over 300 °C), the magnetic phase was still well preserved after calcination at 400 °C. Most importantly, the absence of any Fe^2+^ species on the external surface in all catalysts can be confirmed from XPS spectra (Figure 5).

**Figure 6:**
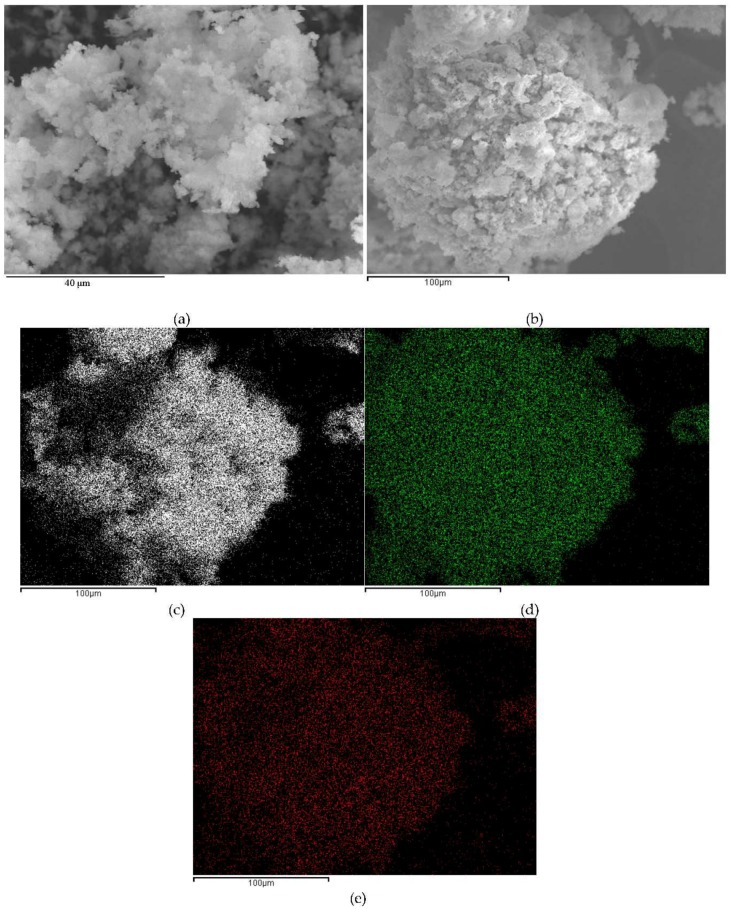
Scanning electron microscopy (SEM) images: (**a**) 5% TiO_2_-MAGSNC photocatalysts; (**b**) 2% TiO_2_-MAGSNC nanocomposites; and elements mapping of 2% TiO_2_-MAGSNC photocatalysts: (**c**) Si; (**d**) Fe; (**e**) Ti.

**Figure 7:**
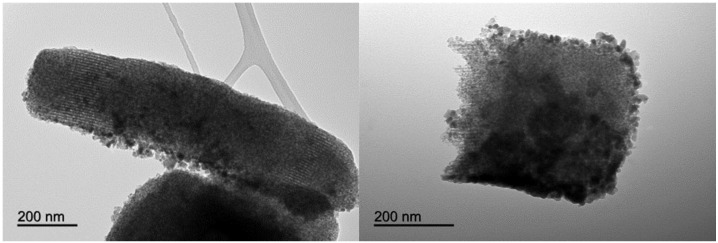
Transmission electron microscopy (TEM) images of 5% TiO_2_-MAGSNC photocatalysts.

Both scanning electron microscopy (SEM) and TEM images of the catalysts were in good agreement with the textural properties and characterization results of the mesoporous nanocomposites (Figure 6 and Figure 7). Element mapping illustrated for 2% TiO_2_-MAGSNC pointed out that both Ti^4+^ and Fe^3+^ were homogeneously distributed on the supports, in line with analogous observations for the other catalysts. Particularly, the fully preserved SBA-15 structure could be visualized in TEM micrographs of the final photocatalytic nanomaterials, with small nanoparticles (*ca.* average nanoparticle size 10 nm), in good agreement with XRD results. Titania nanoparticles could not be distinguished from TEM images, in line with XRD data, which may again relate to a very high dispersion of TiO_2_ in the nanocomposites at such low loadings. Results of EDX analysis have been summarized on Table 2, showing a good agreement in terms of Ti content on the catalysts with respect to the theoretical Ti content selected. These findings confirm the excellent incorporation of Ti provided by the proposed mechanochemical approach.

The magnetic susceptibility of the TiO_2_-MAGSNC photocatalysts were summarized in Table 3, showing that the obtained catalysts all possessed relatively strong ferromagnetism and could be easily separated from the reaction mixture using a simple magnet.

After characterization, the effectiveness of TiO_2_-MAGSNC photocatalysts with different TiO_2_ content was subsequently studied in the photo-oxidation of benzyl alcohol. Photocatalytic activity experiment results have been summarized on Table 4. With illumination time of 4 h, the reaction using TiO_2_-MAGSNC photocatalysts with low TiO_2_ loading (≤1.0 wt %) provided negligible (<5%) photoconversion of benzyl alcohol to benzaldehyde, with conversion only increasing with TiO_2_ loading. The magnetically-separable support (MAGSNC) or SBA-15 itself did not provide any photoactivity under otherwise identical reaction conditions. Bare iron oxides can promote the recombination of photogenerated electron-hole pairs, resulting in inactive materials. Interestingly, a titania loading as low as 5% onto MAGSNC containing the iron oxide phase could significantly decrease the band gap of the TiO_2_ as well as improve the photoconversion of benzyl alcohol (up to 20% in this work) with a remarkable 84% selectivity to the target product. No over-oxidation products, such as benzoic acid and/or CO_2_ from mineralization, were observed in the photo-oxidation of benzyl alcohol photocatalyzed by TiO_2_-MAGSNC. Under the same photocatalytic conditions, the photoconversion of P25 Evonik was obviously quantitative but with an extremely low selectivity to benzaldehyde (32%, over 65% to mineralization), almost comparable in terms of product yield. The enhancement of the photocatalytic properties of the TiO_2_-MAGSNC catalysts, especially in terms of selectivity, makes very attractive this type of magnetically separable nanocomposite containing low titania content, as compared to pure P25 commercial photocatalysts.

## 4. Conclusions

Magnetically-separable catalysts with different content of TiO_2_ were synthesized in a one-pot mechanochemical approach. The synthesized TiO_2_-MAGSNC photocatalysts showed great improvement in light absorption into the visible light range (around 700 nm), with an interesting performance in the photocatalytic conversion of benzyl alcohol to benzaldehyde, particularly at higher loadings (5% Ti). The proposed systems will pave the way to further investigations currently ongoing in our group to the design of photoactive nanomaterials for selective oxidations, which will be reported in due course.

## Figures and Tables

**Table 1:** Optical properties of synthesized TiO_2_-MAGSNC photocatalysts. P25: pure commercial TiO_2_ from Evonik Industries.

Materials	Band Gap [eV]	Absorption Threshold [nm]
TiO_2_-P25	3.21	386
MAGSNC	1.75	705
0.5% TiO_2_-MAGSNC	1.62	765
1.0% TiO_2_-MAGSNC	1.63	761
2.0% TiO_2_-MAGSNC	1.65	751
5.0% TiO_2_-MAGSNC	1.67	740

**Table 2:** Ti and Fe content on TiO_2_-MAGSNC photocatalysts (obtained from energy-dispersive X-ray spectroscopy (EDX) analysis).

Sample ID	Ti (wt %)	Fe (wt %)
0.5% TiO_2_-MAGSNC	0.2	19.2
1.0% TiO_2_-MAGSNC	1.0	24.4
2.0% TiO_2_-MAGSNC	1.7	16.2
5.0% TiO_2_-MAGSNC	4.7	9.6

**Table 3:** The magnetic susceptibility of the TiO_2_-MAGSNC photocatalysts.

Sample ID	Magnetic Susceptibility (× 10^−6^ m^3^·kg^−1^)
0.5% TiO_2_-MAGSNC	116.7
1.0% TiO_2_-MAGSNC	179.1
2.0% TiO_2_-MAGSNC	117.7
5.0% TiO_2_-MAGSNC	130.0

**Table 4:** Photocatalytic oxidation of benzyl alcohol to benzaldehyde ^1^.

Catalyst	Conversion [%]	Selectivity BHA ^2^ [%]	Yield BHA ^3^ [%]
Blank (no catalyst)	-	-	-
SBA-15	-	-	-
MAGSNC	-	-	-
0.5% TiO_2_-MAGSNC	<5	94	-
1.0% TiO_2_-MAGSNC	<5	80	-
2.0% TiO_2_-MAGSNC	<10	73	-
5.0% TiO_2_-MAGSNC	20	84	17
P25 Evonik	>95	32	30

^1^ Reaction conditions: C_o_ benzyl alcohol = 1.5 mM, 125 W lamp, loading: 1 g/L. (solvent: acetonitrile, air flow: 25 mL/min, temperature: 30 °C, reaction time: 4 h). ^2^ BHA: benzaldehyde. ^3^ The selectivity of a reaction was estimated as the ratio of the required product to the undesirable product formed during reaction. Yields were calculated as the ratio of the desired product formed to the total stoichiometric amount. Amount of substance (in mol) were determined using high performance liquid chromatography (HPLC) analysis.

## Abbreviations

The following abbreviations are used in this manuscript:

BA	Benzyl alcohol
BE	Binding energy
BHA	Benzaldehyde
*ca*.	Circa
EDX	Energy-dispersive X-ray spectroscopy
MAGSNC	Magnetically separable nanocomposites
P	Partial vapor pressure of adsorbate gas in equilibrium with the surface at 77.4 K
P_0_	Saturated pressure of adsorbate gas
P25	Pure commercial TiO_2_ from Evonik Industries
SEM	Scanning electron microscopy
TEM	Transmission electron microscopy
UV- Vis	Ultraviolet- Visible
XPS	X-ray photoelectron spectroscopy
XRD	Powder X-ray diffraction
